# Differential Stimulation of Post-Exercise Myofibrillar Protein Synthesis in Humans Following Isonitrogenous, Isocaloric Pre-Exercise Feeding

**DOI:** 10.3390/nu11071657

**Published:** 2019-07-19

**Authors:** Robert W. Davies, Joseph J. Bass, Brian P. Carson, Catherine Norton, Marta Kozior, Miryam Amigo-Benavent, Daniel J. Wilkinson, Matthew S. Brook, Philip J. Atherton, Kenneth Smith, Philip M. Jakeman

**Affiliations:** 1 Department of Physical Education and Sport Sciences, University of Limerick, V94 T9PX Limerick, Ireland; 2 Food for Health Ireland (FHI), Centre for Interventions in Infection, Inflammation and Immunity, University of Limerick, V94 T9PX Limerick, Ireland; 3 Health Research Institute, University of Limerick, V94 T9PX Limerick, Ireland; 4 Medical Research Council (MRC) and Arthritis Research United Kingdom (ARUK) Centre for Musculoskeletal Aging Research and National Institute for Health Research, Nottingham Biomedical Research Centre, University of Nottingham, Nottingham DE22 3DT, UK

**Keywords:** amino acids, deuterium oxide, humans, protein synthesis, resistance training, skeletal muscle, whey proteins

## Abstract

The aim of this study was to test the effects of two disparate isonitrogenous, isocaloric pre-exercise feeds on deuterium-oxide (D_2_O) derived measures of myofibrillar protein synthesis (myoPS) in humans. *Methods:* In a double-blind parallel group design, 22 resistance-trained men aged 18 to 35 years ingested a meal (6 kcal·kg^−1^, 0.8 g·kg^−1^ carbohydrate, 0.2 g·kg^−1^ fat) with 0.33 g·kg^−1^ nonessential amino acids blend (NEAA) or whey protein (WHEY), prior to resistance exercise (70% 1RM back-squats, 10 reps per set to failure, 25% duty cycle). Biopsies of *M. vastus lateralis* were obtained pre-ingestion (PRE) and +3 h post-exercise (POST). The myofibrillar fractional synthetic rate (myoFSR) was calculated via deuterium labelling of myofibrillar-bound alanine, measured by gas chromatography–pyrolysis–isotope ratio mass spectrometry (GC-Pyr-IRMS). Data are a mean percentage change (95% CI). *Results:* There was no discernable change in myoFSR following NEAA (10(−5, 25) %, *p* = 0.235), whereas an increase in myoFSR was observed after WHEY (28 (13, 43) %, *p* = 0.003). *Conclusions:* Measured by a D_2_O tracer technique, a disparate myoPS response was observed between NEAA and WHEY. Pre-exercise ingestion of whey protein increased post-exercise myoPS, whereas a NEAA blend did not, supporting the use of NEAA as a viable isonitrogenous negative control.

## 1. Introduction

Resistance exercise increases uptake of amino acids into the muscle and stimulates muscle protein synthesis (MPS) [1]. An increase in the extracellular concentration of essential amino acids (EAA) [2], particularly leucine [3,4], also stimulates MPS. Thus, consumption of EAA-rich protein in temporal proximity to resistance exercise further stimulates MPS in a dose-dependent manner [5], whereas nonessential amino acids (NEAA) do not have any additional stimulatory effect on MPS when co-ingested with EAA [6,7].

To assess the effect of dietary protein feeding on MPS in a nitrogen-balanced design, an isonitrogenous control is required [8,9]. However, according to authors’ knowledge, the effect of protein ingestion on post-exercise MPS in humans has only been validated against non-nitrogenous (e.g., fasted conditions or a non-caloric placebo) [5,10,11,12] controls, non-nitrogenous isocaloric (e.g., carbohydrate) controls [13,14,15], or isonitrogenous proteins (e.g., whey, casein, and soy) [11,12,16,17], which potentially affect MPS. In a recent ex-vivo cell-based study, we confirmed that a pre-defined blend of NEAA had no effect on de novo protein synthesis [18]. The aim of the present study was to investigate whether the NEAA blend could act as a viable isonitrogenous negative control, in human feeding studies assessing MPS after resistance-exercise. 

In this paper, we report data from a pilot study investigating the effect of pre-exercise NEAA ingestion on post-exercise D_2_O-derived measures of myofibrillar protein synthesis (myoPS) in resistance trained men aged 18 to 35 years. As proof-of-concept, we compared the feeding effects of NEAA to an EAA-rich whey protein (WHEY). We hypothesized that WHEY would stimulate myoPS after resistance exercise [5,7,10,11,12,13,14,15,16], whereas NEAA would not.

## 2. Materials and Methods

### 2.1. Ethical Statement

Prior to enrolling in the study, participants were informed of the risks and benefits associated with participation before providing written informed consent. Ethical approval was granted by the University of Limerick Education and Health Sciences Research Ethics Committee (2016_12_09 EHS, 2013_01_13 EHS), which conforms to standards set by the Declaration of Helsinki. The study was pre-registered at https://www.clinicaltrials.gov as NCT03297151.

### 2.2. Participants

Eligibility criteria were: (i) men aged 18 to 35 years, (ii) six-months of resistance training experience (>3 h·wk^−1^) immediately prior to starting the study, (iii) able to competently perform a 1.25 kg·kg^−1^ body mass one-repetition maximum (1RM) barbell back-squat, (iv) no current injury, illness, medication, or history of chronic disease, and (v) no lactose intolerance. To assess eligibility criteria, a health screen and 1RM test was conducted the week prior to testing.

### 2.3. Study Conduct

**Figure 1:**
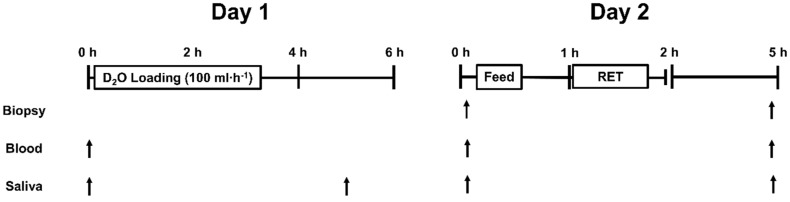
Schematic diagram of the study conduct. D_2_O, deuterium oxide tracer. The isonitrogenous, isocaloric feed was a fixed meal (6 kcal·kg^−1^, 0.8 g·kg^−1^ carbohydrate, 0.2 g·kg^−1^ fat) with a drink containing 0.33 g·kg^−1^ nonessential amino acids or whey protein concentrate/hydrolysate in a randomized double-blind parallel group design.

Participants reported to the laboratory each morning (06:00 to 08:00) fasted overnight (~ 10 h post-absorptive), and refrained from any formal exercise and dietary supplement use for 3 d. Day 1 participants provided saliva and venous blood samples prior to ingesting 400 mL (100 mL·h^−1^) D_2_O (70 atom%). Venous blood was centrifuged (1750 g for 10 min at 22 °C) and plasma was aliquoted and frozen at −80 °C. A second saliva sample was taken 2 h after the final D_2_O bolus (Figure 1). On day 2, a third saliva sample and a micro-biopsy of *m.vastus lateralis* (via standard protocol [19]) was taken to determine basal myofibrillar fraction synthetic rate (myoFSR) (PRE) (Figure 1). Muscle samples were rapidly dissected free of fat and connective tissue, washed in ice-cold saline, snap frozen in liquid N_2_, and stored at −80 °C. Thirty min after the biopsy participants consumed a fixed meal (6 kcal·kg^−1^, 0.8 g·kg^−1^ carbohydrate, 0.2 g·kg^−1^ fat) with a drink containing 0.33 g·kg^−1^ NEAA or whey protein (Table 1). Twenty-four participants were recruited in total and randomly allocated to a whey protein concentrate (WPC, *n* = 8), whey protein hydrolysate (WPH, *n* = 8), or NEAA-group (*n* = 8) in a double-blind parallel group design. Thirty min post-ingestion participants completed a resistance exercise session. A second micro-biopsy of *m. vastus lateralis* and saliva sample was taken 3 h post-exercise (POST) (Figure 1). Two participants (*n* = 1 from each whey protein group) failed to comply with the study conduct and were excluded from the final analysis.

### 2.4. Resistance Exercise

Participants completed sets of 10 barbell back-squats to volitional exhaustion. For the back-squat, participants fixed a loaded barbell (70% 1RM) across the shoulders on the trapezius (above the posterior aspect of the deltoids) and flexed hips and knees until thighs were parallel to the floor, and then extended hips and knees were in an upright standing position [20]. Each repetition was performed at a cadence of 6 s (timed by a metronome or visible timer). Three min rest was taken between sets (25% duty cycle). Volitional exhaustion was operationally defined as the inability to complete a repetition, or an observable change in the technical execution exercise increasing injury risk (e.g., spinal flexion, valgus collapse, asymmetry, and imbalance) [20]. To ensure compliance and form the resistance exercise sessions were supervised by an experienced strength and conditioning professional.

### 2.5. Body Water Enrichment

Pure body water was extracted through heating 100 µL of the saliva sample, before being condensed and transferred to an autosampler vial ready for injection into a high-temperature conversion elemental analyser (TC-EA) (Thermo Finnigan, Thermo Scientific, Hemel Hempstead, UK) connected to an isotope ratio mass spectrometer (IRMS) (Delta V Advantage, Thermo Scientific). To minimise the carryover between samples, each sample was injected four times, with the average of the last three injections used for analysis. For accuracy, a standard curve of known D_2_O enrichment was run alongside samples.

### 2.6. Protein Bound Alanine Enrichment

To measure myofibrillar alanine enrichment, 30 mg of muscle was homogenised in ice cold homogenisation buffer, vortexed for 10 min, and centrifuged at 13,000 g for 10 min at 4 °C before the supernatant was removed. The pellet was solubilized in 0.3 M NaOH before centrifugation at 13,000 *g* for 10 min at 4 °C to separate the insoluble collagen fraction. The myofibrillar containing supernatant was subsequently collected and the proteins were precipitated by the addition of 1 M perchloric acid (PCA). For the plasma proteins, 100 µL of sample was precipitated using 100 µL ice cold ethanol and then separated through centrifugation. Protein-bound amino acids were hydrolysed overnight in 0.1 M HCl and Dowex H^+^ resin at 110 °C, before elution with 2 M NH_4_OH and evaporated to dryness. Amino acids were derivatised to their n-methoxycarbonyl methyl esters by resuspension in 60 µL distilled water and 32 µL methanol, before vortexing and the addition of 10 µL pyridine and 8 µL methylchloroformate. Samples were further vortexed and extracted in 100 µL chloroform and the addition of a molecular sieve to remove any remaining water, before being transferred into a new small volume chromatography vial insert. The deuterium enrichment of protein-bound alanine was measured by sample injection and assessment by gas chromatography–pyrolysis–isotope ratio mass spectrometry (GC-Pyr-IRMS, Delta V Advantage, Thermo Scientific). Samples were injected in triplicate, alongside a standard curve of known L-alanine-2,3,3,3-d4 enrichment.

### 2.7. Calculation of Fractional Synthetic Rate

MyoFSR was calculated from incorporation of deuterium-labelled alanine into the myofibrillar protein using body water as a surrogate for precursor enrichment (corrected for the mean number of deuterium moieties incorporated per alanine (3.7) and the total number of hydrogen atoms within the alanine derivative (11)) [21,22,23]. The equation used is shown below.

(1) $$\mathrm{myoFSR}=-\ln\left(\frac{1-\left[\frac{{\mathrm{APE}}_{\mathrm{Ala}}}{{\mathrm{APE}}_{p}}\right]}{t}\right)$$

where APE_ala_ is deuterium enrichment of protein-bound alanine, APE_p_ is mean precursor enrichment over the study, and t is the time between biopsies.

### 2.8. Postprandial Aminoacidemia

In a separate study, eight participants (age = 25 (5) y, stature = 1.8 (0.1) m, body mass = 82 (12) kg, mean (SD)) were recruited to a double-blind crossover study to assess postprandial aminoacidemia response following isonitrogenous ingestion of the NEAA blend, whey protein concentrate (WPC), and whey protein hydrolysate (WPH) (0.33 g·kg^−1^ body mass). In this study, participants reported to the lab on three separate occasions, at least 4 d apart, (06:00 to 08:00) fasted overnight (~10 h post-absorptive), refrained from any formal exercise, and nutritional supplement use 3 d prior to testing. A cannula was inserted into the antecubital vein and a blood sample was collected in EDTA s-monovette^®^ tubes (PRE). Participants then ingested 0.33 g·kg^−1^ of each test drink (Table 1) and postprandial blood samples were collected at 30 min intervals, up to 180 min. Participants remained in a seated position throughout the sampling period. Blood was centrifuged (e.g., 514× *g*, 4 °C for 10 min, Eppendorf 5417R, Eppendorf AG, Hamburg, Germany) plasma was aliquoted and stored at −80 °C for analysis. 

Plasma amino acid profile was measured (via a standard protocol [24]), using the Agilent 1200 RPUPLC system (Agilent Technologies, Santa Clara, CA, USA) equipped with an Agilent 1260 binary pump and a G1367C automated liquid handling system. Chemstation software (Agilent Technologies Inc.) was used for data acquisition. Separation of amino acids was carried out using a C_18_ ZORBAX rapid resolution column (4.6 × 50 mm, 1.8 µm, Agilent Technologies Inc.) thermo-stated at 40 °C. Quantitative analysis was performed by the external standard method, using the Agilent amino acid standard (Agilent Technologies Inc.). Standards were measured in both not spiked and spiked pool plasma and recoveries were calculated. Total area under the curve (AUC) was calculated using the trapezoidal rule. 

### 2.9. Statistical Analysis

Descriptive statistics are mean (SD). Change (Δ) and percent change (Δ%) values (PRE to POST) are reported mean (95% CI). For statistical analysis, normality and homogeneity were confirmed prior to performing parametric statistical tests. Mixed-model ANOVA was used to assess group × time interaction. Independent *t*-tests were used to assess difference between groups. Paired *t*-tests were used to assess a difference within the groups. The critical significance level was α = 0.05. A false-discovery rate correction was used to adjust for a familywise error [25]. Sample size estimates (1 − β = 0.8) were calculated from previously published data [22]. The magnitude of the change was examined by effect size calculation (Cohen’s d [26]). In areas where there was no difference between the WPC and WPH groups (data provided Appendix A), data were pooled into a single WHEY-group (*n* = 14) for comparative evaluation against the NEAA (*n* = 8). Statistical tests were performed in RStudio (1.1.383).

## 3. Results

### 3.1. Participants and Resistance Training Performance

No differences were observed between NEAA and WHEY groups for age, stature, body mass, 1RM strength, resistance training experience, or resistance training performance (Table 2).

### 3.2. Aminoacidemia 

#### 3.2.1. Plasma Amino Acid Concentration

**Figure 2:**
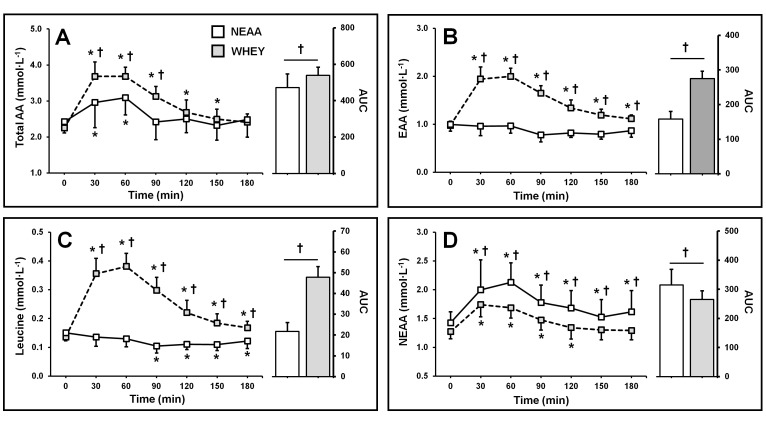
Postprandial plasma amino acid (AA) (Panel **A**), essential amino acid (EAA) (Panel **B**), leucine (Panel **C**), and nonessential amino acid (NEAA) concentration (Panel **D**) following isonitrogenous ingestion of a NEAA blend (open, *n* = 8) or whey protein (grey, *n* = 14). Data are mean +/− SD. † significant difference between groups. * Significant difference from baseline (0 min) (*p* < 0.05).

Plasma amino acid concentration AUC was 16 (11) % higher in the WHEY vs. NEAA-group (*p* = 0.003). There was a group × time interaction (*p* < 0.001). Amino acid concentration was higher in the WHEY-group vs. NEAA at 30, 60, and 90 min (*p* < 0.012). Postprandial hyperaminoacidemia was observed up to 60 min in the NEAA-group (*p* < 0.034) and up to 150 min in the WHEY-group (*p* < 0.004) (Figure 2A).

#### 3.2.2. Plasma Essential Amino Acid Concentration

EAA AUC was 76 (17) % higher in the WHEY vs. NEAA-group (*p* < 0.001). A group × time interaction was observed (*p* < 0.001). Postprandial EAA concentration was higher in the WHEY-group vs. NEAA-group at all time-points (*p* < 0.001). No increase in EAA was observed in the NEAA-group (*p* > 0.255), whereas postprandial increases were observed up to 180 min in the WHEY-group (*p* < 0.005) (Figure 2B).

#### 3.2.3. Plasma Leucine Concentration

Plasma leucine AUC was 124 (28) % higher in the WHEY vs. NEAA-group (*p* < 0.001). A group × time interaction was observed (*p* < 0.001). Postprandial leucine concentration was higher in the WHEY-group vs. NEAA-group at all time-points (*p* < 0.001). In the NEAA group, leucine concentration decreased below PRE after 90 min (*p* < 0.001), whereas postprandial increases in leucine were observed up to 180 min in the WHEY-group (*p* < 0.002) (Figure 2C).

#### 3.2.4. Plasma Nonessential Amino Acid Concentration

Plasma NEAA AUC was 19 (10) % higher in the NEAA vs. WHEY-group (*p* < 0.001). A group × time interaction was observed (*p* < 0.001). Total NEAA concentration was higher in the NEAA-group vs. WHEY-group at all time-points (*p* < 0.05). Postprandial increases were observed up to 180 min in the NEAA-group (*p* < 0.034) and up to 120 min in the WHEY-group (*p* < 0.004) (Figure 2D).

### 3.3. Body Water Enrichment

Body D_2_O water enrichment increased 0.49 (0.06) % PRE (WHEY 0.49 (0.05) % vs. NEAA 0.51 (0.08) %, *p* = 0.456) and was stable through the 5 h study period (0.49 (0.06) % POST, *p* = 0.772).

### 3.4. Myofibrillar Protein Synthesis

**Figure 3:**
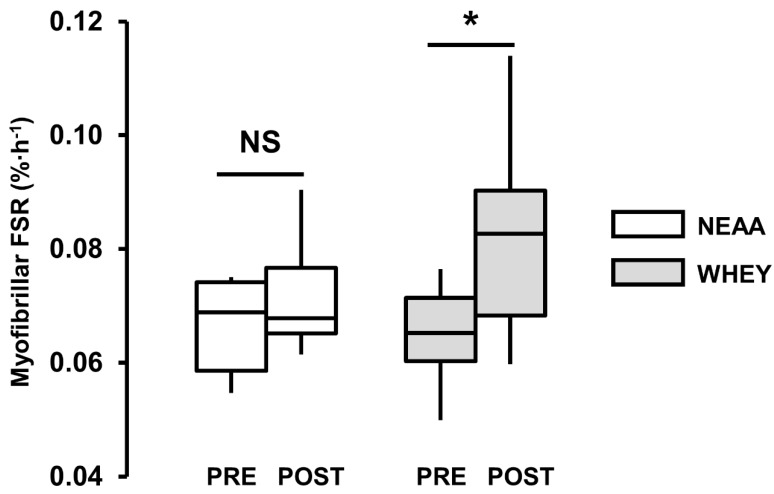
Myofibrillar fractional synthetic rate (FSR, %·h^−1^) pre-ingestion (PRE) and 3 h post-exercise (POST). Isonitrogenous whey protein group (WHEY, *n* = 14, open boxes) and nonessential amino acid group (NEAA, *n* = 8, grey boxes). Boxplot shows median (line) and interquartile range (box), whiskers represent the maximum and minimum values. * Significant difference vs. PRE; NS, not-significantly different vs. PRE (*p* < 0.05). Group × time interaction (*p* = 0.059).

There was no myoFSR difference between groups PRE (WHEY 0.065 (0.008) %·h^−1^ vs. NEAA 0.066 (0.009) %·h^−1^, *p* = 0.717). Group differences were observed PRE to POST, but failed to reach the critical significance level (Δ = 0.011 (0.006, 0.016) %·h^−1^, d = 0.7, *p* = 0.059). There was no myoFSR change in the NEAA-group (POST = 0.072 (0.010) %·h^−1^, Δ = 0.006 (-0.003, 0.014) %·h^−1^, Δ% = 10 (–5, 25) %, d = 0.4, *p* = 0.235), whereas a myoFSR increase was observed in the WHEY-group (POST = 0.082 (0.016) %·h^−1^, Δ = 0.017 (0.008, 0.026) %·h^−1^, Δ% = 28 (13, 43) %, d = 0.9, *p* = 0.003) (Figure 3).

## 4. Discussion

In this paper, we report data from a pilot study investigating the effects of two isonitrogenous, isocaloric pre-exercise feeds on post-exercise, D_2_O-derived, measures of myoPS in resistance trained men aged 18 to 35 years. One feed contained 0.33 g·kg^−1^ NEAA blend, while the other contained WHEY. In line with previous reports, we observed a 28% increase in myoPS with WHEY [5,7,10,11,12,13,14,15,16], and report no significant change in post-exercise myoPS after ingestion of NEAA. We and others have previously employed the D_2_O tracer technique assess basal, postprandial, and post-exercise myoFSR in humans [21,22,23], but this is the first study to use the D_2_O method to evaluate the combined effects of feeding and resistance exercise on acute (<5 h post-exercise) measures of myoPS.

The amino acid profile of the NEAA blend, used in the present study, was modelled on the NEAA composition of milk protein (Table 1). Postprandial aminoacidemia response following WHEY and NEAA blend ingestion was confirmed, at rest, in a separate study (Figure 2). In this study, hyperaminoacidemia was observed in both groups (NEAA + 0.6 mmol·L^−1^ ~ 40%, WHEY + 1.6 mmol·L^−1^ ~ 70%), which peaked 60 minutes post-ingestion. However, following WHEY ingestion, there was a robust increase in the plasma concentration of the key nutrient regulators of myoPS (i.e., EAA (+1.1 mmol ~ 75%) and leucine (+0.23 mmol ~ 190%)). We conducted a preliminary evaluation of bioactivity of the NEAA blend in a cell-based study, where the myotube culture was conditioned with participants’ postprandial serum [18]. No change in de novo protein synthesis, phosphorylation of mTOR, p70S6k, or 4e-BP1 was observed in the NEAA condition. Conversely, increases in de novo protein synthesis and anabolic signaling were observed after WHEY [18].

In humans, NEAA do not appear to have any additional stimulatory effect on post-exercise MPS when co-ingested with EAA [6,7]. However, to our knowledge, the independent effect of NEAA ingestion (i.e., without EAA) on post-exercise myoPS has not been verified. In the present human study, the basal (0.06 (0.01) %·h^−1^) and postprandial/postexercise myoFSR (0.08 (0.02) %·h^−1^) was comparable to other human studies employing similar exercise/feeding interventions [5,23,27,28]. Thus, the finding that pre-exercise ingestion of NEAA evoked no increase post-exercise myoPS, supports the passive role for NEAA in the regulation of myoPS/MPS [6,7,18], and promotes the potential role of the NEAA blend as an isonitrogenous negative control for the measurement of myoPS in protein feeding studies. Comparative evaluation of dietary protein (or derivative) ingestion against an isonitrogenous, isocaloric control in a nitrogen-balanced design, provides a more accurate and comprehensive appraisal of the potential bio-efficacy of proprietary proteins or protein derivatives, promoted for supplemental use in clinical or athletic populations. Therefore, as proof-of-concept, we compared the effect of the NEAA (negative control) to an EAA-rich whey-protein (positive control). The myoFSR increase after WHEY, confirms the sensitivity of D_2_O-derived measures of myoPS to detect an acute protein feeding response (<5 h), and further supports the efficacy of the NEAA blend as a viable negative control for the measurement of myoPS in protein feeding studies [11,12,16].

Based on our knowledge, this is the first study to report acute stimulation of post-exercise D_2_O-derived myoPS following ingestion of a reputable dietary protein supplement, known to enhance post-exercise myoPS [5,7,10,11,12,13,14,15,16]. Recently, it has been discussed whether interventions that increase short-term lab-based measures of myoPS/MPS can be used to enhance muscle remodeling/hypertrophy over a longer period of time [29,30]. One advantage of the D_2_O tracer technique is the relatively slow decay of the precursor pool (i.e., body water) [22], which allows continuous measurement of free-living myoPS to be made over days [21] or weeks [21,31]. Therefore, future research can employ the D_2_O tracer method to assess the effect of supplemental dietary protein on longer-term measures of myoPS.

## 5. Conclusions

In conclusion, we demonstrate that pre-exercise ingestion of two disparate isonitrogenous, isocaloric feeds, containing NEAA and WHEY, differentially effect post-exercise myoPS. Pre-exercise ingestion of EAA-rich WHEY increased post-exercise D_2_O-derived measures of myoPS, while ingestion of an isonitrogenous NEAA blend (0.33 g·kg^−1^) did not appear to stimulate myoPS. The findings from the present study supports the passive role for NEAA in the regulation of myoPS, and promotes the use of NEAA as a viable isonitrogenous negative control in human feeding studies investigating the role of dietary protein on myoPS.

## Figures and Tables

**Table 1:** Amino acid composition of the whey protein and nonessential amino acid blend.

	WHEY		NEAA	
	mg·kg^−1^	%	mg·kg^1^	%
Alanine	17	5	33	10
Arginine	8	2	0	0
Aspartic acid	35	11	41	12
Cysteine	8	2	0	0
Glutamic acid	56	17	120	36
Glycine	6	2	13	4
Histidine	6	2	0	0
Isoleucine	20	6	0	0
Leucine	34	10	0	0
Lysine	30	9	0	0
Methionine	7	2	0	0
Phenylalanine	10	3	0	0
Proline	19	6	53	16
Serine	17	5	45	14
Threonine	23	7	0	0
Tryptophan	7	2	0	0
Tyrosine	9	3	25	8
Valine	19	6	0	0
EAA	0.16	48	0	0
NEAA	0.17	52	0.33	100
Total	0.33		0.33	

Isonitrogenous whey protein (WHEY) and nonessential amino acid (NEAA) blend dose was scaled to a body mass (kg) concentrate and hydrolysate amino acid composition was equivalent. Data are mg·kg^−1^ body mass and % total mass.

**Table 2:** Baseline characteristics.

	NEAA (*n* = 8)	WHEY (*n* = 14)	*p*-Value
Age (y)	23 (5)	23 (4)	0.666
Stature (m)	1.77 (0.04)	1.82 (0.07)	0.120
Body mass (kg)	77 (17)	81 (10)	0.455
1RM (kg·kg^−1^)	1.5 (0.3)	1.5 (0.2)	0.985
RET experience (y)	2.6 (1.5)	2.4 (1.1)	0.602
RET performance (sets)	8.7 (1.5)	7.6 (1.8)	0.287

Data are mean (SD). 1RM, barbell back-squat one repetition maximum per kg body mass. RET, resistance exercise training.

## Appendix A

	**NEAA (*n* = 8)**	**WPC (*n* = 7)**	**WPH (*n* = 7)**
myoFSR PRE (%·h^−1^)	0.066 (0.008)	0.068 (0.006)	0.063 (0.008)
myoFSR POST (%·h^−1^)	0.072 (0.010)	0.084 (0.017)	0.079 (0.016)
% change	10 (−5, 25)	27 (2, 51) *	30 (5, 55) *

Myofibrillar protein fractional synthetic rate (myoFSR) data pre-ingestion (PRE) and 3 h post-exercise (POST). NEAA, nonessential amino acid group. WPC, whey protein concentrate group. WPH, whey protein hydrolysate group. Data are mean (SD) or (95% CI). *p* = 0.377 (3 × 2 ANOVA). * Significant difference vs. PRE (*p* < 0.05) (paired *t*-test).
